# Resident factors associated with American board of internal medicine certification exam failure

**DOI:** 10.1080/10872981.2022.2152162

**Published:** 2022-11-28

**Authors:** Preston H. Seaberg, Juliana M. Kling, Molly C. Klanderman, Carolyn Mead-Harvey, Kathryn E. Williams, Helene R. Labonte, Atul Jain, Gretchen E. Taylor, Janis E. Blair

**Affiliations:** a Department of Internal Medicine Charleston Division, West Virginia University School of Medicine, Charleston, West Virginia, USA; b Division of Women’s Health Internal Medicine, Mayo Clinic, Scottsdale, Arizona, USA; c Division of Clinical Trials and Biostatistics, Mayo Clinic, Scottsdale, Arizona, USA; d Pulmonary/Sleep Medicine, ProMedica Physicians, Toledo, Ohio, USA; e Division of Community Internal Medicine, Mayo Clinic, Scottsdale, Arizona, USA; f Division of General Internal Medicine, Mayo Clinic, Scottsdale, Arizona, USA; g Division of Hospital Internal Medicine, Mayo Clinic, Phoenix, Arizona, USA; h Division of Infectious Diseases, Mayo Clinic, Phoenix, AZ, USA

**Keywords:** Internal medicine, graduate medical education, internship and residency, american board of internal medicine, learner assessment, physician certification, computer-based assessment, physician certifying examination, board examination, standardized test

## Abstract

**Introduction:**

Performance on the certifying examinations such as the American Board of Internal Medicine Certification Exam (ABIM-CE) is of great interest to residents and their residency programs. Identification of factors associated with certification exam result may allow residency programs to recognize and intervene for residents at risk of failing. Despite this, residency programs have few evidence-based predictors of certification exam outcome. The change to pass-or-fail score reporting of the USA Medical Licensing Exam (USMLE) Step 1 removes one such predictor.

**Materials and Methods:**

We performed a retrospective study of residents from a medium-sized internal medicine residency program who graduated from 1998 through 2017. We used univariate tests of associations between ABIM-CE result and various demographic and scholastic factors.

**Results:**

Of 166 graduates, 14 (8.4%) failed the ABIM-CE on the first attempt. Failing the first attempt of the ABIM-CE was associated with older median age on entering residency (29 vs 27 years; *P* = 0.01); lower percentile rank on the Internal Medicine In-Training Examination (IM-ITE) in each of the first, second, and third years of training (*P* < 0.001 for all); and lower scores on the USMLE Steps 1, 2 Clinical Knowledge, and 3 (*P* < 0.05 for all). No association was seen between a variety of other scholastic or demographic factors and first-attempt ABIM-CE result.

**Discussion:**

Although USMLE step 1 has changed to a pass-or-fail reporting structure, there are still other characteristics that allow residency programs to identify residents at risk of ABIM-CE first time failure and who may benefit from intervention.

## Introduction

Passing a certification exam – commonly referred to as a board exam or simply ‘the Boards’ – is an important milestone for the residency program graduate. Residency program staff also have an abiding interest in their graduates’ board exam performance. For example, beyond wanting graduates to succeed, residency programs must achieve specific aggregate certifying exam pass rates to meet the Common Program Requirements of the Accreditation Council for Graduate Medical Education (ACGME) [1]. For internal medicine residency programs, for example, a 3-year rolling average first-attempt board exam pass rate of 80% or greater is required to meet accreditation standards. Identifying factors associated with exam performance can help programs ensure both graduate success and ongoing program accreditation.

There is a gap between which factors educators expect to be linked to board exam performance and which associations have empirical evidence to support such a linkage. For example, in a 2013 survey, residency program directors proposed links between internal medicine board exam performance and various factors such as ACGME work hour requirements, performance on other standardized tests, teaching conference attendance, and competing personal obligations [2]. Published data to test such hypotheses are scant but reliably show that higher scores on the USA Medical Licensing Examination (USMLE) [3–6] and the Internal Medicine In-Training Examination (IM-ITE) [3–7] are associated with a higher likelihood of passing the ABIM-CE. No association has been found between board exam performance and work hour requirements [8,9] or competing personal obligations like parenting or recent life stressors[10]. Investigators have found similar relationships in the fields of surgery and radiology, where written board exam performance is associated with performance on the USMLE [11,12] or respective in-training exam [13–15], with no association between surgical board exam performance and work hour requirements [16]. Teaching conference attendance may be associated with IM-ITE performance [17] but is not independently associated with ABIM-CE performance [18].

Within internal medicine, research regarding associations between passing the ABIM-CE and participation in specific clinical experiences is inconclusive [3,19,20], whereas participation in a mandatory research year in general surgery residency was associated with passing American Board of Surgery General Surgery Certifying Exam [13]. Incorporating the timing of rotations with overnight call, however, investigators at the Cleveland Clinic developed a nomogram to determine an internal medicine resident’s probability of passing the ABIM-CE using IM-ITE scores and number of months with overnight call in the final 6 months of residency [3]. Other investigators have examined links between ABIM-CE performance and characteristics including age [3,10], gender [3,10,21], time elapsed between medical school graduation and residency [3], fellowship interest [10], and scholarly productivity [22]. No durable associations have been found between these individual factors and board exam performance.

Single-center studies on this topic have examined associations over a short time span, and multicenter studies have tested for associations in only 1 year. Instruments used by faculty members to assess resident performance (e.g., ABIM Monthly Evaluation Form and ABIM Resident Evaluation Form are no longer used) have changed over time, and certain factors such as the content, frequency and length of required educational conferences vary by institution. The current study aimed to use data from a longitudinal sample to identify factors durably associated with first-attempt ABIM-CE performance among internal medicine residents from a medium-sized residency program in the USA.

## Materials and Methods

We performed a retrospective study of all residents who graduated from the Internal Medicine Residency Program at Mayo Clinic in Arizona from 1998 through 2017. The program has full ACGME accreditation, and its size gradually increased from an initial 6 residents per class to its current 12 residents per class. The program is affiliated with a tertiary care hospital in a large metropolitan area in the Southwest USA. Residents in the program during the study period were provided access to ABIM-CE preparation resources, including the Medical Knowledge Self-Assessment Program, MedStudy Internal Medicine Board Review, and the opportunity to participate in a funded, off-site ABIM-CE preparatory course. Residents with IM-ITE performance below a prespecified score were enrolled in an individualized education plan with access to another question bank and check-ins from an associate program director. The study protocol was approved by the Institutional Review Board of the Mayo Clinic in Arizona (application 13–000080). The Board conducted a risk-benefit analysis, determined the study constituted minimal risk research, and waived the requirement to obtain informed consent.

Residency program coordinators, the principal investigator, and a nonmedical volunteer abstracted demographic and scholastic data from archived files of all program graduates into a digital spreadsheet. Information was deidentified by these abstractors through substitution of a number for the graduate’s name, the key for which was kept by the principal investigator. Information collected and subsequently coded as binary variables included gender, marital status, primary language, medical degree type (e.g., MD, DO, MBBS, or MB, BCh), off-cycle residency graduation, Alpha Omega Alpha (AOA) Honor Medical Society membership, childrearing or childbearing in residency, graduation from a medical school outside the USA, graduate or professional degrees earned before starting residency, completing a rotation at the program’s institution before starting residency, board review course completion, a gap of at least 1 year between completion of medical school and starting residency, area of program director concern during residency, and publication of an article or abstract during residency. Information collected on work hour requirements was coded as a categorical variable, with specific requirements noted in Table 1. Data collected on the following factors were coded as continuous variables: USMLE scores, IM-ITE percentile ranks, distance of home address from program address, time separating medical school and residency graduation, and age on starting residency. Distance of a resident’s home address from the program’s street address was determined using the shortest driving route between the 2 locations according to Google Maps.

**Table 1.** ACGME Work Hour Requirements.

‘Pre-2003 Requirements’ included all of the following: Limit of 80 hours of work per week, when averaged over four weeks^[23]^ Minimum of one day in seven free from scheduled activities, averaged over four weeks^[23]^ Maximum frequency of in-house call of once every three nights, averaged over four weeks^[23]^ “2003–2011 Requirements” included all of the following: All three “pre-2003 requirements”^[23]^ Adequate rest period, ideally a minimum of 10 hours, between duty periods^[23]^ Limit of 24 hours of continuous duty, with up to 6 added hours for education and continuity of care^[23]^ Option to request an increase of up to 8 hours to the weekly duty hour limit, if certain criteria met^[24]^ “Post-2011 Requirements” included all of the following: All three “pre-2003 requirements”^[24]^ Minimum of 8 hours time off between scheduled duty periods, with very limited exceptions^[24]^ Limit of 24 hours of continuous duty (16 hours for first-year residents), with up to 4 added hours for continuity of care, with very limited exceptions^[24]^ 6 nights maximum of consecutive in-house night float duty^[24]^ Option to request an increase of up to 8 hours to the weekly duty hour limit, if certain criteria met^[24]^

We used univariate analysis to test associations between various resident factors and first-attempt ABIM-CE results classified as pass or fail. Binary and categorical variables are presented as counts and percentages. Continuous variables are presented as median (range or interquartile range). Associations were tested by using Fisher’s exact test for categorical variables and Wilcoxon rank sum test for continuous variables, and correlations were estimated with the Pearson correlation coefficient. *P* < 0.05 was the threshold for statistical significance. R Statistical Software version 3.6.2 was used to conduct the analysis.

## Results

Among 167 residents, 1 did not graduate and was excluded from analysis. Data from 166 graduates were included for analysis. One resident restarted the residency program after a leave of absence, and data from work before the leave of absence were excluded. The overall first-attempt board exam failure rate of graduates was 8.4% (n = 14). Resident demographic characteristics are summarized in Table 2.

**Table 2.** Characteristics by result of first attempt at board exam^a.^

	Board exam pass		
Characteristic	No (n = 14)	Yes (n = 152)	*P* value
Age at start of residency, y	29.0 (28–31.8)	27.0 (26–29)(n = 151)	.01
Time between medical school graduation and residency graduation, y	3.0 (3–3)	3.0 (3–3)	.88
Distance of home address from program address, miles	13.7 (13.0–19.4)(n = 13)	13.8 (8.7–19.8)(n = 150)	.66
Gender = Woman	6 (42.9)	78 (51.3)	.59
MD (vs DO, MBBS, MB, BCh)	13 (92.9)	148 (97.4)	.36
Chief resident year	2 (14.3)	31 (20.4)	.74
Pursued subspecialty practice after graduation	11 (78.6)	88 (57.9)	.16
Alpha Omega Alpha Membership	0 (0.0)	29 (19.1)	.13
Marital status, single	6 (42.9)	67 (44.1)	>.99
Parented during residency	3 (21.4)	46 (30.3)	.76
Gave birth during residency	1 (7.1)	18 (11.8)	>.99
Institutional rotation during medical school	3 (21.4)	25 (16.4)	.71
Graduated from medical school outside USA	0 (0.0)	16 (10.5)	.37
Gap between medical school graduation and starting residency	2 (14.3)	22 (14.5)	>.99
Work hours			.46
2003–2011	9 (64.3)	76 (50.0)	
Post-2011	3 (21.4)	30 (19.7)	
Pre-2003	2 (14.3)	46 (30.3)	
Additional graduate or professional degree	1 (25.0)(n = 4)	12 (27.3)(n = 44)	>.99
Primary language other than English	1 (7.1)	12 (7.9)	>.99

Abbreviations: DO, doctor of osteopathic medicine; MB, BCh, bachelor of medicine, bachelor of surgery; MBBS, bachelor of medicine, bachelor of surgery; MD, doctor of medicine.

^a^Values are median (interquartile range) or No. of residents (%).

Compared with graduates who failed the board exam on the first attempt, graduates who passed on the first attempt started residency at a significantly younger median [interquartile range] age (no pass: 29 [28–31.8] years vs pass: 27 [26–29] years; *P* = 0.01) (Table 1). There were no significant between-group differences among the other examined demographic factors (Table 2).

Graduates who passed the board exam on the first attempt had higher performance on standardized tests taken before and during residency, including median percentile ranks on the IM-ITE in each of the first (63 vs 36), second (71 vs 32), and third (69 vs 24.5) years of residency (*P* < 0.001 for all) (Table 3). Similarly, those who passed the ABIM-CE on the first attempt had higher median USMLE scores for Step 1 (222 vs 206; *P* = 0.02), Step 2 Clinical Knowledge (230 vs 210; *P* = 0.01), and Step 3 (221 vs 200; *P* < 0.001). There were no between-group differences in other scholastic performance factors evaluated (Table 3).

**Table 3.** In-Residency scholastic factors by result of first attempt at board exam.

	Board exam pass^a^		
Factor	No (n = 14)	Yes (n = 152)	*P* value
Area of program director concern present during residency	3 (21.4)	15 (9.9)	.18
Off-cycle residency graduation date	2 (14.3)	4 (2.6)	.08
Published article in residency	10 (71.4)	99 (65.1)	.77
Published abstract in residency	13 (92.9)	139 (96.5)(n = 144)	.43
Attended board review course	10 (90.9)(n = 11)	81 (85.3)(n = 95)	>.99
IM-ITE percentile rank			
PGY1	36.0 (28.0–43.5)(n = 10)	63.0 (47.0–82.0)(n = 133)	<.001
PGY2	32.0 (11.0–39.0)(n = 13)	71.0 (52.2–85.0)(n = 142)	<.001
PGY3	24.5 (12.0–58.8)	69.0 (50.0–85.0)(n = 148)	<.001
USMLE score			
Step 1	206.0 (204–218)(n = 13)	222.0 (208.5–232)(n = 151)	.02
Step 2 CK	210.0 (193.5–225)(n = 11)	230.0 (216.5–244)(n = 147)	.01
Step 3	200.0 (198–212)(n = 11)	221.0 (211–233)(n = 139)	<.001

Abbreviations: CK, Clinical Knowledge; IM-ITE, Internal Medicine In-Training Examination; PGY, postgraduate year; USMLE, USA Medical Licensing Examination.

^a^Values are median (interquartile range) or No. of residents (%).

We next analyzed the correlation between age and IM-ITE and USMLE scores. Pearson correlation coefficients ranged from −0.07 to −0.22 (Table 4). The correlation was significant between age and postgraduate year 2 IM-ITE score (*r* = −0.19; *P* = 0.02), postgraduate year 3 IM-ITE score (*r* = −0.22; *P* = 0.005), and USMLE Step 3 score (*r* = −0.18; *P* = 0.03).

**Table 4.** Correlation Between Residents’ Test Scores and Age at the Start of Residency.

Variable	Estimate (95% CI)	Statistic	*P* value
IM-ITE percentile rank			
PGY1	–0.07 (–0.23 to 0.09)	–0.85	.39
PGY2	–0.19 (–0.34 to – 0.04)	–2.42	.02
PGY3	–0.22 (–0.36 to – 0.07)	–2.87	.005
USMLE score			
Step 1	–0.15 (–0.30 to 0.00)	–1.94	.06
Step 2 CK	–0.15 (–0.30 to 0.01)	–1.91	.06
Step 3	–0.18 (–0.33 to – 0.02)	–2.22	.03

Abbreviations: CK, Clinical Knowledge; IM-ITE, Internal Medicine In-Training Examination; PGY, postgraduate year; USMLE, USA Medical Licensing Examination.

## Discussion

In our study population, residents who passed the ABIM-CE on the first attempt had higher performance on the USMLE and IM-ITE than did peers who failed the ABIM-CE. This is in line with findings of other investigators [3–7]. Unlike in other investigations, however, we found that residents who passed the ABIM-CE on the first attempt also were younger at the start of residency than those who failed. Potential explanations for the correlation in our population between increasing age and decreasing USMLE Step 3 and IM-ITE scores are not readily apparent.

We hypothesized that among residents in our study population, those entering residency at younger ages may have different average test scores than those entering at older ages. Further analysis identified a small negative correlation between age and some of the IM-ITE and USMLE scores. This suggests that those who were younger on entering residency had slightly higher exam scores during residency than did those who started residency at older ages. Residents who were older may be more likely to have more family commitments – marriage, childbearing, childrearing, or aging family members with caregiver needs – and competing time demands outside of work. However, those who were married, who bore children or who raised children were not significantly older on average than residents who were single and did not bear or raise children (28.7 years vs. 28.3 years, *P* = 0.47). Furthermore, there was no significant difference in the proportion of residents with such additional family commitments who passed the first-attempt ABIM-CE and those who failed (59.9% vs. 57.1%, *P* = 1.00). Future research with larger sample sizes and wider-ranging demographic data collection could help determine why an association might exist between certifying examination performance and age at residency entry.

Burnout is associated with adverse physician and perhaps even patient outcomes [25], but data linking certification status to burnout are scarce. An association between burnout and ABIM-CE result makes intuitive sense. That adequate social support would decrease likelihood of developing burnout also makes intuitive sense, though data are conflicting. While Ripp and co-investigators [26] reported no association between certain social supports and incident resident burnout, Ironside and colleagues [27] reported residents perceive social relationships to be protective against burnout. Further, Salles and others [28] reported an inverse association between social belonging and thoughts of leaving surgical residency. We did not design our study to test for an association between burnout and ABIM-CE performance. Following data collection, we hypothesized that residents who graduated from in-state medical schools might have more numerous local social ties and more robust social support networks, contributing to a higher likelihood of passing the ABIM-CE through lower rates of loneliness-associated conditions such as burnout. However, a lower proportion of our residents who passed the ABIM-CE graduated from an in-state medical school, though this difference was not statistically significant (17.8% vs. 28.6%, *P* = 0.30). Future studies with dedicated measurement of burnout would be valuable to determine whether an association exists between burnout and certifying exam performance.

Additional factors that may potentially associate with board passage include AOA membership and post-residency fellowship pursuit. Of residents who passed the ABIM-CE in our cohort, a higher proportion were AOA members (19.1% vs. 0.0%), and a lower proportion pursued subspecialty practice after residency graduation (57.9% vs. 78.6%), though these differences were not statistically significant. Horn and colleagues [29] in radiology and Shellito and others [30] in general surgery found associations between resident AOA membership and passing results on their respective board exams. Grossman et al. evaluated those enrolled in fellowship programs when taking the ABIM-CE for the first time in 1991 were significantly more likely to pass the exam than were those not in fellowship[10]. There are methodologic differences that do not allow direct comparison of our fellowship-trained graduates and those in the study by Grossman et al, [10] namely that in our study some of our graduates took the ABIM-CE before entering fellowship.

Our study’s strength was in inclusion of novel factors not examined in the existing internal medicine literature. These included AOA membership, completion of an audition rotation at our hospital while in medical school, and an estimate of commute distance. It also included results from data collected over a longer period than in other studies, a characteristic that might mitigate potentially confounding effects of broader changes in residency training and the ABIM-CE over time. Finding differences that do – or do not – endure over time may make those findings more reliable or externally valid.

We acknowledge study limitations, including the size of the study. With the given ABIM-CE pass rate, the study was not powered to reach statistical significance for between-group differences of small magnitudes. The study is exploratory in nature, and to reduce type II error, no corrections were made for multiple. All findings require validation. Another limitation is that the study population includes residents at only 1 center. Residents in our residency program may not be representative of those in another, or of those in other specialties. Importantly, we did not collect data that reflected the program’s active efforts to increase the ABIM-CE pass rate. These efforts included implementing interventions such as medical knowledge enhancement opportunities for residents whose IM-ITE scores were concerning for failing the boards, and programs to systematically ensure all residents’ review of relevant topics. For instance, in 2019, the residency program created and offered protected time for third-year residents to attend a program-created weekly review of medicine topics and practice tests for those in the last six months of residency, with a 100% first-attempt ABIM-CE pass rate in the three years since.

In addition, other than including the updated ACGME work hour rules, our study does not account for institutional factors that may influence resident performance. As examples, investigators have found possible associations between resident-specific ABIM-CE results and timing of certain clinical experiences [3], number of specific clinical experiences [19,20], and performance in a specific ambulatory curriculum [31]. Additionally, residency program–level ABIM-CE pass rates may be associated with a program’s institutional classification (e.g., university program, university-affiliated community program), size, and location [2,32,33]; length of a program director’s tenure in that role [2]; program-level resident demographics [2]; faculty-to-resident ratio [2,33]; amount of external graduate medical education funding [34]; ACGME survey results [35]; and ACGME Residency Review Committee certification length [32]. Knowledge of such factors could facilitate the creation of program-level interventions to offset the effects of individual factors associated with lower likelihood of passing the ABIM-CE.

Lastly, as illustrated by the change in USMLE Step 1 score reporting, some of these variables may not be useful in the future. Critical appraisal of current assessment methods and markers of achievement may identify systemic issues, even if explanations for such issues can only be theorized. As an example, in the Summary Report and Preliminary Recommendations from the March 2019 Invitational Conference on USMLE Scoring (InCUS), Barone and others [36] noted racial differences in USMLE performance that persisted despite examination content monitoring. Wijesekera and colleagues [37] also found gender and racial disparities in which residency program applicants inducted into one or both of two honor societies. Decision-making in medical education should incorporate the highest quality empirical evidence as well as ethical principles. If a given measure of performance or achievement proves ineffective or conflicts with ethical principles, it should be removed from use.

## Conclusion

The USMLE Step 1 score reporting has changed from a numeric score to pass or fail[38]. Over time, evaluation instruments provided by the ACGME and specialty boards and used by residency program faculty members to assess resident performance have also changed. Accordingly, within internal medicine, association of faculty member assessment of resident performance and ABIM-CE result has been variable across time, with some investigators finding an association [39–43] and others finding none [44]. These points introduce uncertainty into attempts at the timely identification of residents at risk of failing their respective certifying examinations. Novel methods of assessment are needed to better predict performance on standardized measures of competence or achievement. The results of this study and others like it may help educators identify and deliver targeted interventions for residents at risk of failing the ABIM-CE, primarily those with low performance on standardized examinations before and during residency and those who started residency at an older age.

## Data Availability

Due to the nature of this research, participants of this study did not agree for their data to be shared publicly, so supporting data are not available.
